# The Coexistence of Microscopic Polyangiitis and Rheumatoid Arthritis: A Case Report

**DOI:** 10.7759/cureus.63885

**Published:** 2024-07-05

**Authors:** Claudia S Villa Celi, Valeria Turcan, Juan Sosa, Natalia Plotskaya, Manish Gugnani

**Affiliations:** 1 Internal Medicine Residency Program, Capital Health Regional Medical Center, Trenton, USA; 2 Internal Medicine, Capital Health Regional Medical Center, Trenton, USA; 3 Pulmonary and Critical Care Medicine, Capital Health Regional Medical Center, Trenton, USA

**Keywords:** bronchoalveolar lavage (bal), positive ana, mpo-anca, anca associated vasculitis, pauci-immune crescentic glomerulonephritis, diffuse alveolar hemorrhage, rheumatoid arthitis, microscopic polyangiitis (mpa)

## Abstract

Microscopic polyangiitis (MPA) is a rare autoimmune disease characterized by the inflammation and necrosis of small vessels, primarily affecting kidneys and lungs. It is classified as an antineutrophil cytoplasmic antibody (ANCA)-associated vasculitis (AAV) due to the presence of ANCA. MPA can manifest as diffuse alveolar hemorrhage (DAH) and rapidly progressive glomerulonephritis. In contrast, rheumatoid arthritis (RA) is an inflammatory disease that mainly targets the synovial joints. The coexistence of these two conditions presents significant diagnostic challenges, highlighting the need for further research and understanding.

We report a case of a 58-year-old male with a past medical history of RA, chronic bronchitis, tobacco use, and recent Legionella pneumonia who presented with acute dyspnea. The patient was intubated for acute hypoxemic respiratory failure. Laboratory workup revealed anemia, hyponatremia, and acute kidney injury. Urinalysis showed hematuria and proteinuria. A CT scan of the chest exhibited bilateral extensive patchy infiltrates. He was transfused with one packed red blood cell (PRBC) unit. Hemoglobin decreased below 6 g/dL after transfusion. A bronchoscopy revealed erythema throughout the tracheobronchial tree, and blood on bronchial alveolar lavage suggested DAH. High-dose steroids were started. Subsequent laboratory results were positive for rheumatoid factor (RF), perinuclear ANCA (p-ANCA), anti-myeloperoxidase (anti-MPO), and antinuclear antibody (ANA). The kidney biopsy demonstrated focal crescentic necrotizing glomerulonephritis pauci-immune type, confirming MPA.

RA pathogenesis involves immune dysregulation and activation of various cells, leading to the release of cytokines. Antibodies such as RF and anti-cyclic citrullinated peptide (anti-CCP) can be detected up to 10 years before the clinical manifestation of RA. Recent studies have revealed a predominance of MPA in AAV while coexisting with RA. The underlying mechanism of its occurrence remains unclear. Our patient had recurrent respiratory symptoms and renal dysfunction before hospitalization. MPA-RA overlap syndrome is potentially treatable and clinicians should maintain a high index of suspicion when encountering patients with preexisting RA. Timely initiation of immunosuppressive therapy at early stages is essential to prevent renal and pulmonary complications. ANCA serology should be assessed in these cases.

## Introduction

Microscopic polyangiitis (MPA) is a type of antineutrophil cytoplasmic antibody (ANCA)-associated vasculitis (AAV) characterized by necrotizing small-vessel vasculitis without granulomatous inflammation, predominantly affecting the kidneys and lungs. MPA can manifest as diffuse alveolar hemorrhage (DAH) and as rapidly progressive glomerulonephritis [1]. Rheumatoid arthritis (RA), on the other hand, is a chronic autoimmune disease primarily targeting the joints but can involve multiple organs [2]. While each condition independently poses significant challenges, the coexistence of MPA and RA presents a unique diagnostic and therapeutic dilemma due to overlapping clinical features [3].

We present a case report highlighting the rarity of MPA alongside RA, diagnosed in a 58-year-old male patient. We aim to emphasize the complexities of diagnosing and managing these autoimmune conditions through clinical evaluation, diagnostic workup, and treatment.

## Case presentation

A 58-year-old male patient with a past medical history of RA, chronic kidney disease (CKD), and recurrent productive cough with hoarseness for three months presented to the hospital complaining of acute dyspnea. Notably, six months ago, the patient had been diagnosed with Legionella pneumonia and acute kidney injury. Upon arrival, the patient was in respiratory distress. He was initially placed on a nonrebreather mask but transitioned to bilevel-positive airway pressure (BiPAP) due to declining oxygen saturation. Vital signs on presentation were as follows - blood pressure: 130/74 mmHg, heart rate: 97 beats per minute, respiratory rate: 20 breaths per minute, and oxygen saturation: 100% on BiPAP. Bilateral coarse rales were appreciated on auscultation. 

Laboratory investigations showed anemia with a hemoglobin level of 6.6 g/dl, leukocytosis (12.31 x 10^3^/μL with no bandemia), electrolyte imbalances including hyponatremia (sodium: 133 mmol/L), hypokalemia (serum potassium: 2.8 mmol/L), metabolic acidosis (chloride: 109 mmol/L, bicarbonate 15 mmol/L), acute renal failure with a blood urea nitrogen (BUN) of 27 mg/dL, and creatinine (Cr) 2.03 mg/dl (baseline Cr was 1.2 mg/dl), and elevated inflammatory markers with an erythrocyte sedimentation rate (ESR) higher than 150 mm/hr and procalcitonin level of 1.58 ng/mL. Urinalysis demonstrated both hematuria and proteinuria.

**Figure 1:**
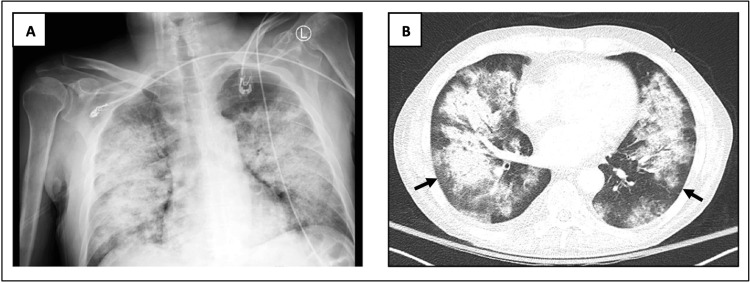
X-ray (A) and CT scan (B) of the chest on admission The images showed bilateral pulmonary infiltrates sparing the periphery (arrows) CT: computed tomography

Imaging studies, including chest X-ray and CT, revealed bilateral extensive patchy infiltrates sparing the periphery (Figure 1).

**Figure 2:**
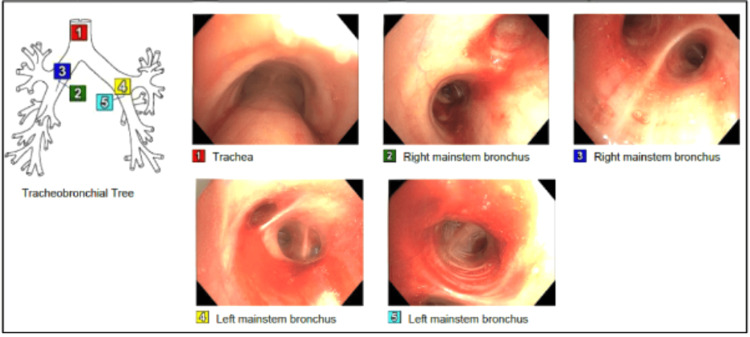
Bronchoscopy Bronchoscopy showed erythema throughout the tracheobronchial tree and bloody fluid on bronchial alveolar lavage (BAL) aspiration

The patient was given a blood transfusion and started empirically on levofloxacin for suspected Legionella infection. However, despite the blood transfusion and no signs of active bleeding, the hemoglobin continued to decline, necessitating further investigation. Bronchoscopy revealed diffuse erythema throughout the tracheobronchial tree and bloody fluid on bronchial alveolar lavage (BAL) aspiration, consistent with DAH (Figure 2). High-dose intravenous methylprednisolone (300 mg every eight hours) was promptly initiated.

**Figure 3:**
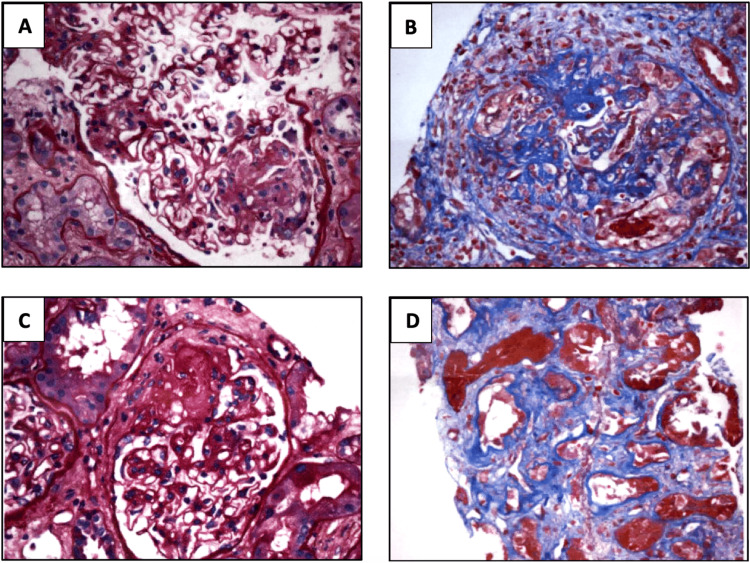
Kidney biopsy sections stained with H&E, PAS, trichrome, and JMS (magnification 20x) A. Cellular crescent B. Fibrinoid necrosis C. Fibrous crescent D. Interstitial fibrosis Kidney biopsy demonstrated focal necrotizing and diffuse crescentic glomerulonephritis, pauci-immune type with mild activity and moderate chronicity. Tubular atrophy and interstitial fibrosis were moderate

Subsequent investigations were conducted to elucidate the underlying etiology. Tests for Legionella antigen, blood cultures, and several autoimmune markers were negative. On the other hand, the rheumatoid factor (RF) was elevated at 24.4 U/mL, the antinuclear antibody (ANA) was positive, and elevated anti-myeloperoxidase (MPO) antibodies were shown to suggest AAV. Given the pulmonary-renal involvement and laboratory findings, a kidney biopsy was recommended, which confirmed the diagnosis of AAV, specifically MPA, characterized by focal crescentic necrotizing glomeruli, 40% fibrosis of the glomeruli, acute as well as chronicity noted, and the absence of granulomas (Figure 3).

The patient completed five days of high-dose methylprednisolone and seven days of levofloxacin. He was successfully extubated on day seven of hospitalization. He was then started on rituximab infusion and a steroid taper. The patient's symptoms and radiographic findings improved, with no oxygen requirement and Cr level back to baseline. He was discharged on day 14 and scheduled for a rheumatology follow-up and a weekly rituximab infusion for four weeks.

## Discussion

RA is a chronic autoimmune inflammatory disorder that mainly affects the synovium but can have extra-articular involvement. Laboratory workup shows elevated levels of ESR, CRP, RF, and anti-cyclic citrullinated peptide (anti-CCP). It requires early treatment to prevent degrease progression and increased mortality and morbidity [2]. AAV is a rare autoimmune condition that causes inflammation of the small blood vessels. It includes granulomatosis with polyangiitis (GPA; previously known as Wegener granulomatosis), eosinophilic granulomatosis with polyangiitis (EGPA; previously known as Churg-Strauss syndrome), and MPA [1,4].

GPA is characterized by necrotizing granulomatous inflammation of the respiratory tract, pauci-immune crescentic glomerulonephritis, and positivity for cytoplasmic-ANCA (c-ANCA). Eosinophilia, necrotizing granulomatous inflammation, and asthma are characteristics of EGPA. MPA is distinctive for non-granulomatous necrotizing vasculitis, mainly affecting the lungs and kidneys. EGPA and MPA are typically positive for perinuclear-ANCA (p-ANCA) [4-6]. The coexistence of MPA and RA presents a rare but significant clinical scenario. Both are autoimmune diseases characterized by chronic inflammation, usually targeting different organs and with distinct immunopathogenic mechanisms. However, some studies have shown that AAV and RA share the same genetic features, such as protein tyrosine phosphatase non-receptor type 22 (PTPN22), polymorphism in uteroglobin, and nuclear factor kappa-light-chain-enhancer of activated B cells (NF-κB2) [3].

Our patient presented with respiratory distress secondary to DAH and worsening kidney function, a hallmark manifestation of MPA, possibly associated with exacerbation of his underlying RA as evidenced by the presence of RF and ANA [7]. A multidisciplinary approach encompassing clinical, radiological, serological, and histopathological assessments and interdisciplinary collaboration was imperative to establish a diagnosis. Given the patient's recent history of Legionella, leukocytosis, and increased inflammatory markers, along with CT scan findings, Legionella pneumonia was initially suspected, prompting the commencement of antibiotic therapy. However, the rapid respiratory deterioration, the peripheral sparing in the CT, the persistence of anemia, and negative infectious workup necessitated further investigation, eventually leading to the diagnosis of MPA [5,8-10]. He received a combination of high-dose corticosteroids, rituximab, and supportive measures tailored to both MPA and RA. The treatment was successful, leading to clinical improvement, resolution of respiratory distress, and kidney function returning to baseline.

## Conclusions

The pathogenesis of RA involves immune dysregulation and activation of various cells, leading to the release of cytokines. Antibodies such as RF and anti-CCP can be detected up to 10 years before the clinical manifestation of RA. Recent research has revealed a predominance of MPA in AAV while coexisting with RA. While the underlying mechanism of its occurrence remains unclear, it represents a diagnostic and therapeutic challenge. Our patient had recurrent respiratory symptoms and renal dysfunction before hospitalization. MPA-RA overlap syndrome is potentially treatable and clinicians should have a high index of suspicion when they encounter patients with preexisting RA. Timely initiation of immunosuppressive therapy at early stages is essential to prevent renal and pulmonary complications.
